# Cardiorenal protective effects of Tanhuo decoction in acute myocardial infarction via regulating multi-target inflammation and metabolic signaling pathways

**DOI:** 10.3389/fphar.2025.1555605

**Published:** 2025-03-27

**Authors:** Chenglong Guo, Tianxing Zhang, Lingqian He, Minyu Zhang, Yanyan Chu, Xipeng Sun, Xuexue Han, Yijiang Liu, Juexian Song, Jinggang Xia

**Affiliations:** ^1^ Pulmonary Vascular Disease Center, Beijing Anzhen Hospital, Capital Medical University, Beijing, China; ^2^ Department of Cardiology, Xuanwu Hospital, Capital Medical University, Beijing, China; ^3^ Department of Neurology, Xuanwu Hospital, Capital Medical University, Beijing, China; ^4^ School of Traditional Chinese Medicine, Capital Medical University, Beijing, China; ^5^ Senior Department of Hepatology, The Fifth Medical Center of PLA General Hospital, Beijing, China

**Keywords:** acute myocardial infarction, Tanhuo decoction, cardiorenal protection, inflammation, metabolism

## Abstract

**Introduction:**

Inflammation is a key driver of adverse outcomes in acute myocardial infarction (AMI), yet current western anti-inflammatory therapies are limited by their single-target nature and side effects. Traditional Chinese medicine (TCM), such as Tanhuo Decoction (THD), offers a multi-target, low-toxicity alternative.

**Methods:**

In a randomized controlled trial, AMI patients with high inflammatory responses received either standard Western medicine (WM) alone or combined with THD for 3 days. Clinical outcomes and inflammatory markers were assessed, and proteomic and network pharmacology analyses were performed.

**Results:**

The THD + WM group showed significant reductions in neutrophil counts and hs-CRP levels, along with improved creatinine clearance rate (CCR), compared to WM alone. Proteomic analysis revealed downregulation of pro-inflammatory proteins (PTX3, IL-18, TNFRSF11A) and upregulation of the anti-inflammatory IL1RL2. THD also modulated lipid metabolism and insulin sensitivity pathways.

**Discussions:**

THD enhances the anti-inflammatory and metabolic benefits of standard AMI therapy through multi-target pathway regulation. These findings support its integration into modern cardiovascular care, particularly for patients with high inflammatory and metabolic risk.

## 1 Introduction

Acute myocardial infarction (AMI) represents the most severe manifestation of coronary atherosclerotic heart disease. Despite significant advancements in pharmacological treatments and revascularization techniques in recent years, the incidence of AMI continues to rise annually. In-hospital mortality rates for AMI remain around 8%, while heart failure occurs in 14%–36% of patients during hospitalization (Wu et al., 2019; Chan et al., 2020). This underscores the importance of AMI as a pressing public health challenge.

Coronary atherosclerosis is a chronic inflammatory condition fueled by cholesterol deposition, with inflammation linked to metabolic disorders such as obesity and diabetes, identified as key contributors to what is now recognized as “residual inflammatory risk” (Weber et al., 2023; Priscilla et al., 2024). In recent years, research focusing on targeting different inflammatory pathways has garnered substantial attention within the cardiovascular and metabolic fields. Several studies have demonstrated that mitigating inflammation can significantly reduce the risk of recurrent adverse cardiovascular events in AMI patients, offering a new and promising perspective on the clinical management of AMI (Ridker et al., 2023). Some landmark clinical trials in the field of anti-inflammatory therapy for atherosclerotic cardiovascular disease have provided robust evidence for the clinical application of anti-inflammatory treatments. These include the CANTOS trial (Maffia and Guzik, 2019), the COLCOT trial (Roubille et al., 2024), and the LoDoCo2 trial (Opstal et al., 2022), all of which have advanced the clinical relevance of anti-inflammatory approaches in treating atherosclerosis. However, Western anti-inflammatory drugs often target singular pathways, come with high costs, and carry the risk of adverse effects. For example, IL-1β monoclonal antibodies have not been widely adopted in clinical practice due to their association with increased risk of fatal infections (Matter et al., 2024). Currently, only low-dose colchicine (0.5 mg/day) has been recommended for secondary prevention of coronary artery disease in the 2023 ESC Guidelines for Acute Coronary Syndromes (Byrne et al., 2023). Nonetheless, concerns remain regarding its side effects such as gastrointestinal reactions, pneumonia, and the potential for increased non-cardiovascular mortality.

In recent years, traditional Chinese medicine (TCM) has increasingly been recognized for its systemic regulatory effects, offering a unique multi-target approach that both directly and indirectly modulates signaling pathways. These characteristics make it particularly effective in reducing inflammation and improving outcomes in the treatment of coronary heart disease (Yang et al., 2023; Ge et al., 2021). Tanhuo decoction (THD), a traditional formula developed from years of clinical practice, has shown notable efficacy in the acute phase of ischemic stroke (Guo et al., 2021). Clinical research has demonstrated that THD significantly improves outcomes in patients with acute cerebral infarction. It enhances cerebral perfusion, reduces serum levels of inflammatory cytokines, inhibits the inflammatory response associated with acute stroke, and even regulates gut microbiota, contributing to overall recovery and health maintenance (Guo et al., 2022). This study employed a randomized controlled trial involving 40 patients with AMI to evaluate the effectiveness of THD in reducing inflammation and enhancing patient outcomes. Additionally, the research aimed to explore the potential mechanisms underlying these effects, providing a robust evidence base for the application of TCM in the treatment of AMI. Through this approach, the study seeks to offer further empirical support for integrating TCM into modern cardiovascular care.

## 2 Methods

### 2.1 Study design and oversight

This study employed a prospective randomized controlled design, targeting patients with AMI of high inflammatory response who presented within 12 h of onset. Participants were randomly assigned into a Tanhuo Decoction combined with western medicine treatment group (WM + THD) and a western medicine treatment group (WM), with 20 eligible patients selected for each group. The randomization in this study was conducted using a random number table method. Each of the 40 enrolled patients was assigned a unique identification number. A pre-generated random number table was used to allocate patients into two groups: the WM + THD group and the WM group, with 20 patients in each. Odd-numbered entries from the random number table were assigned to one group, while even-numbered entries were assigned to the other, ensuring an unbiased and balanced distribution. This research, which adhered to the standards outlined in the Declaration of Helsinki and followed the principles of Good Clinical Practice, received approval from the Xuanwu Hospital Ethics Committee under document number 2022–129. All enrolled patients signed informed consent forms.

### 2.2 Study population

All enrolled patients were recruited from Xuanwu Hospital, Capital Medical University, between April 2024 and October 2024. Enrollment criteria included patients aged from 18 to 80 who presented within 12 h of symptom onset with type I AMI, along with HSCRP levels ≥3 mg/L. The diagnosis of AMI was established based on the Fourth Universal Definition of Myocardial Infarction, which requires evidence of myocardial injury, indicated by a rise and/or fall in cTn levels, with at least one measurement exceeding the 99th percentile of the upper reference limit (URL). Additionally, at least one of the following criteria must be met: symptoms of acute myocardial ischemia, new ischemic ECG changes, pathological Q-wave formation, imaging findings of regional wall motion abnormalities or loss of viable myocardium consistent with ischemic pathology, or the presence of a coronary thrombus confirmed by angiography. Exclusion criteria extended to those with severe complications related to AMI, such as mechanical issues, persistent cardiogenic shock unresponsive to vasopressors, uncontrolled acute left heart failure or pulmonary edema, and malignant arrhythmias not manageable with antiarrhythmic drugs at the time of admission. Furthermore, patients with severe comorbid conditions, including major hepatic or renal dysfunction, serious infections, bleeding predisposition, cancers, or a projected life expectancy under 1 year, were not eligible. Additionally, patients with hypersensitivity to metabolites of THD were formally excluded.

### 2.3 Procedures

All patients underwent percutaneous coronary intervention (PCI) surgery and received standard western medical treatment, including antiplatelet therapy (aspirin 100 mg/day, clopidogrel 75 mg/day), anticoagulation therapy (low molecular weight heparin 100 AxaIU/kg every 12 h for 3 days), and lipid-lowering therapy (atorvastatin 20 mg/day). Patients in the WM + THD group consumed one daily 200 mL dose of the decoction, divided into two portions taken in the morning and evening, for a period of 3 days. Each unit of THD contained 9 g of Coptidis Rhizoma, 5 g of Rhei Radix et Rhizoma, 9 g of Forsythia, 9 g of Lophatherum gracile, and 9 g of Bile Arisaema. The botanical drugs, sourced from Sinopharm Group Beijing Huamiao Pharmaceutical Co., Ltd., adhered to the standards set by the China Pharmacopoeia (2015 edition). Quality control was conducted by the supplier, who also identified the primary compounds through high-performance liquid chromatography. The batch number and the content of the main chemicals of each botanical drug were listed in Supplementary Table S1. Xuanwu Hospital, Capital Medical University, prepared the decoction in line with standard production protocols. Additionally, fire-heat scores were employed to assess symptoms associated with fire-heat syndromes, such as constipation, dry mouth, yellowish urine, and a reddened tongue (Guo et al., 2021).

### 2.4 Blood sample

Peripheral venous blood was collected from admitted AMI patients using ethylenediamine tetraacetic acid (EDTA) anticoagulant tubes. Following collection, samples were centrifuged at 3,000 rpm and 1,650 g for 15 min. The separated plasma was then carefully transferred to Eppendorf tubes and stored at −80°C for preservation.

### 2.5 Proteomics

Proteins were measured using the Olink^®^ Target 96 Cardiovascular III (v.6114) panel (Olink Proteomics AB, Uppsala, Sweden) according to the manufacturer’s instructions. The Proximity Extension Assay (PEA) technology used for the Olink protocol has been well described (Assarsson et al., 2014) and enables 92 analytes to be analyzed simultaneously, using 1 µL of each sample. In brief, pairs of oligonucleotide-labeled antibody probes bind to their targeted protein, and if the two probes are brought in close proximity the oligonucleotides will hybridize in a pair-wise manner. The addition of a DNA polymerase leads to a proximity-dependent DNA polymerization event, generating a unique PCR target sequence. The resulting DNA sequence is subsequently detected and quantified using a microfluidic real-time PCR instrument (Signature Q100, LC-Bio Technology CO., Ltd., Hangzhou, China). Data is then quality controlled and normalized using an internal extension control and an inter-plate control, to adjust for intra- and inter-run variation. The final assay read-out is presented in Normalized Protein eXpression (NPX) values, which is an arbitrary unit on a log2-scale where a high value corresponds to a higher protein expression. All assay validation data (detection limits, intra- and inter-assay precision data, *etc.*) are available on manufacturer’s website (www.olink.com).

### 2.6 Analysis of network pharmacology

#### 2.6.1 THD bioactive metabolite collection and target gene prediction

The bioactive metabolites of THD were collected from the TCM system pharmacology database and analysis platform (TCMSP, tcmspw. com/tcmsp.php), with oral bioavailability (OB) ≥ 30% and drug-like properties (DL) ≥ 0.18. Chemical structures were retrieved through the PubChem database (pubchem.ncbi.nlm.nih.gov) following literature review of THD bioactive metabolites. Target genes potentially regulated by these metabolites were predicted using Swiss Target Prediction (www.swisstargetprediction.ch) (Kim et al., 2021; Daina et al., 2019). The UniProt database (www.uniprot.org) was utilized to standardize gene names, with filtering conditions set to “Reviewed” and “*Homo sapiens* (Human) (Uniprot, 2023).

#### 2.6.2 Acquisition of AMI targets and network establishment

Disease-related targets of AMI were identified through multiple databases, including GeneCards (www.genecards.org), DrugBank (www.drugbank.com), Disease Gene Search Engine (DigSee, 210.107.182.61/geneSearch), Online Mendelian Inheritance in Man (OMIM, omim.org), and DISGENET (www.disgenet.org), using the search terms “Acute Myocardial Infarction,” “Acute Myocardial Infarct,” and “Cardiovascular Stroke” (Amberger et al., 2015; Stelzer et al., 2016; Wishart et al., 2018). The retrieved AMI-related target genes were standardized using the UniProt database (Uniprot, 2023). A network illustrating the relationships among drugs, active metabolites, and therapeutic targets of THD was constructed using Cytoscape 3.8.2 (Shannon et al., 2003). Protein-protein interaction (PPI) networks for THD targets and AMI disease targets were independently established using String within Cytoscape, with species restricted to “*H. sapiens*” and a minimum interaction score threshold of 0.4 (Xu et al., 2018). These networks were subsequently merged to identify common targets between THD and AMI for further analysis. The molecular complex detection (MCODE) plugin in Cytoscape was employed to classify the common targets into highly correlated protein clusters. Analysis parameters were set as follows: Degree Cutoff = 2, Node Score Cutoff = 0.2, K-Core = 2, and Max. Depth = 100. The cluster with the highest MCODE score, which correlates positively with gene criticality within the network, was identified as the core protein complex mediating THD’s therapeutic effects on AMI.

#### 2.6.3 Gene ontology (GO) and kyoto encyclopedia of genes and genomes (KEGG) enrichment analysis

The core protein cluster metabolites were analyzed using the Metascape database (www.metascape.org) for GO enrichment analysis, encompassing GO Biological Process (BP), GO Molecular Function (MF), and GO Cellular Metabolite (CC), and KEGG enrichment analysis (Liu et al., 2011). Analysis parameters included a minimum overlap of 3, P-value cutoff of 0.01, and minimum enrichment of 1.5. Results from GO and KEGG enrichment analyses were visualized graphically (Chen et al., 2011).

### 2.7 Statistics

Baseline data were analyzed using GraphPad Prism 8. Continuous variables were reported as mean ± standard deviation (SD) or median with interquartile range (IQR), while categorical variables were shown as frequencies. Depending on the distribution of continuous variables, either Student’s t-test or the Mann-Whitney U test was applied for comparisons. Categorical variables were compared using the chi-square test. ROC curve analysis was performed using a logistic regression model, with the area under the curve (AUC) calculated accordingly. A p-value below 0.05 was deemed statistically significant.

## 3 Results

### 3.1 Clinical characteristics

**FIGURE 1:**
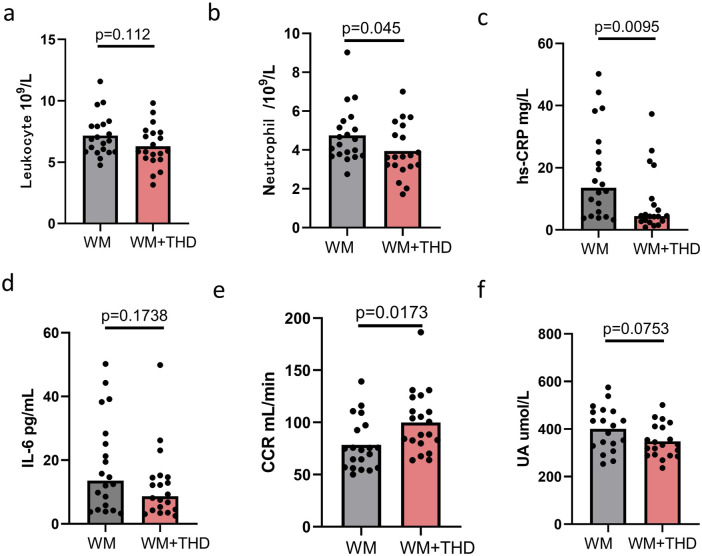
Effects of WM and WM + THD on inflammatory markers, renal function, and metabolic parameters. **(A)** Leukocyte count: No significant difference between WM and WM + THD groups (p = 0.112); **(B)** Neutrophil count:WM + THD treatment significantly reduced neutrophil levels compared to WM (p = 0.045); **(C)** hs-CRP: WM + THD significantly decreased hs-CRP levels compared to WM (p = 0.0095); **(D)** IL-6: No significant difference between WM and WM + THD groups (p = 0.1738); **(E)** CCR: WM + THD significantly improved CCR compared to WM (p = 0.0173p = 0.0173p = 0.0173). **(F)** UA: A trend toward reduced UA levels in the WM + THD group compared to WM, though not statistically significant (p = 0.0753). Data are presented as mean ± standard error of the mean (SEM). Statistical significance was determined by p < 0.05.

A total of 40 patients were enrolled and randomly assigned to the THD + WM group and the WM group, with 20 patients in each group. There were no significant differences in baseline clinical characteristics between the two groups, as shown in Table 1. As illuminated in Table 2 and Figure 1, after 3 days of treatment, patients in the WM + THD group demonstrated significantly lower neutrophil counts (3.945 ± 0.442 × 10^9^/L VS 4.751 ± 0.345 ×10^9^/L, P = 0.045) and hs-CRP levels (8.621 ± 3.967 mg/L VS 18.25 ± 3.865 mg/L, P = 0.0095) compared to the WM group, as well as a significant increase in creatinine clearance rate (CCR) (100 ± 8.67 mL/min VS 78.38 ± 7.46 mL/min, P = 0.0173). Additionally, there was a noticeable trend toward lower uric acid levels, although this difference was not statistically significant (348.3 ± 26.39 μmol/L VS 400.8 ± 26.39 μmol/L, P = 0.0753). The sensitive indicators of liver injury such as alanine aminotransferase (ALT) and aspartate aminotransferase (AST) showed no significant elevation compared with the two groups. Moreover, patients experienced no gastrointestinal adverse effects such as nausea or vomiting, indicating that THD is safe and well-tolerated without causing gastrointestinal complications.

**TABLE 1:** Clinical Characteristics of AMI patients in WM and WM + THD treatment groups at baseline.

Row name	WM N = 20	WM + THD N = 20	*p-*Value
Sex, male (%)	16 (80)	16 (80)	1.000
Age, y (mean [SD])	64 ± 11	63 ± 10	0.564
BMI, kg/m2 (mean [SD])	26.8 ± 3.8	25.2 ± 1.4	0.077
Current smoker, yes (%)	11 (55)	8 (45)	0.527
History of hypertension, yes (%)	13 (65.0)	14 (70.0)	0.736
History of diabetes, yes (%)	8 (40.0)	8 (40.0)	1.000
Type of AMI, n (%)			
STEMI	17 (85.0)	13 (65.0)	0.144
NSTEMI	3 (15.0)	7 (35.0)	0.144
GRACE risk score (mean [SD])	163 ± 32	157 ± 31	0.532
TIMI risk score (median [IQR])	4 (3–5)	3 (2–4)	0.052
Blood glucose, mmol/L (median [IQR])	7.13 (6.31–9.55)	6.34 (5.31–7.99)	0.149
HbA1c, % (median [IQR])	5.8 (5.5–7.3)	6.0 (5.4–7.8)	0.795
CCR, mL/min (median [IQR])	84.73 (60.67–105.83)	95.73 (76.55–116.31)	0.327
TC, mmol/L (median [IQR])	4.34 (3.97–4.97)	4.36 (3.92–4.94)	0.613
LDL-C, mmol/L (mean [SD])	2.77 ± 0.69	2.63 ± 0.73	0.548
HDL-C, mmol/L (mean [SD])	1.02 ± 0.25	1.04 ± 0.28	0.883
Triglycerides, mmol/L (median [IQR])	1.76 (1.16–2.13)	1.39 (1.11–1.96)	0.377
Serum uric acid, mmol/L (mean [SD])	353 ± 100	400 ± 81	0.111
Peak troponin I, ng/mL (median [IQR])	22.70 (8.28–37.40)	6.70 (2.65–34.70)	0.212
NT-pro BNP, pg/mL (median [IQR])	489 (106–2,222)	673 (165–2,779)	0.863
hs-CRP, mg/L (median [IQR])	7.83 (3.55–24.66)	8.65 (4.00–11.97)	0.758
IL-6, pg/mL (median [IQR])	20.47 (11.83–29.44)	19.17 (13.66–33.44)	0.841
Leukocyte,10^9^/L (median [IQR])	9.95 (7.94–11.05)	8.94 (7.07–11.12)	0.429
NEUT#,10^9^/L (median [IQR])	8.21 (6.50–9.13)	7.27 (4.74–9.26)	0.414
Cardiac function killip degree, n (%)			0.303
Killip 1	12 (60.0%)	16 (80.0%)	
Killip 2	7 (35.0%)	4 (20.0%)	
Killip 3	1 (5.0%)	0 (0.0%)	
LVEF, % (mean [SD])	53 ± 9	55 ± 8	0.491

BMI, body mass index; AMI, acute myocardial infarction; STEMI, ST-segment–elevation myocardial infarction; NSTEMI, non–ST-segment–elevation myocardial infarction; GRACE, global registry of acute coronary events; TIMI, thrombolysis in myocardial infarction; HbA1C, glycosylated hemoglobin, type A1C; CCR, creatinine clearance rate; TC, total cholesterol; LDL-C, low-density lipoprotein cholesterol; HDL-C, high-density lipoprotein cholesterol; NT-proBNP, N-terminal-pro-B-type natriuretic peptide; hs-CRP, high-sensitivity C-reactive protein; IL-6, interleukin-6; NEUT, neutrophil count; LVEF, left ventricular ejection fraction; SD, standard deviation and IQR, interquartile range.

**TABLE 2:** Clinical Characteristics of AMI patients in WM and WM + THD groups after 3 days treatment.

Row name	WM N = 20	WM + THD N = 20	*p-*Value
Blood glucose, mmol/L (median [IQR])	5.13 (4.83–6.29)	5.19 (5.26–5.71)	0.149
ALT, IU/L (median [IQR])	45.75 (20–172)	52 (21–110)	0.531
AST, IU/L (median [IQR])	35.4 (12–88)	38.85 (17–80)	0.547
CCR, mL/min ((mean [SD])	78.38 ± 7.46	100 ± 8.67	0.0173
TC, mmol/L (mean [SD])	3.66 ± 1.04	3.35 ± 0.54	0.252
LDL-C, mmol/L (mean [SD])	2.10 ± 0.82	1.74 ± 0.41	0.089
HDL-C, mmol/L (mean [SD])	0.91 ± 0.20	0.93 ± 0.22	0.724
Triglycerides, mmol/L (mean [SD])	1.46 ± 0.56	1.43 ± 0.45	0.834
Serum uric acid, mmol/L (mean [SD])	400.8 ± 26.39	348.3 ± 26.39	0.0753
hs-CRP, mg/L (mean [SD])	18.25 ± 3.865	8.621 ± 3.967	0.0095
IL-6, pg/mL (median [IQR])	10.39 (7.45–20.68)	8.69 (4.34–14.65)	0.173
Leukocyte,10^9^/L (median [IQR])	7.17 (7.00–16.77)	6.30 (4.28–15.07)	0.112
NEUT#,10^9^/L (mean [SD])	4.751 ± 0.345	3.945 ± 0.442	0.045

ALT, alanine aminotransferase; AST, aspartate aminotransferase.

Furthermore, Supplementary Figure S1 illustrates that patients treated with WM for 3 days experienced an upward trend in hs-CRP (15.72 ± 4.817 mg/L VS 18.25 ± 3.865 mg/L, P = 0.786) and uric acid levels (352.6 ± 30.92 μmol/L VS 400.8 ± 26.39 μmol/L, P = 0.1269) instead of a reduction. In contrast, as depicted in Supplementary Figure S2, 3 days of WM + THD treatment resulted in a marked decrease in both hs-CRP (13.85 ± 3.568 mg/L VS 8.621 ± 3.967 mg/L, P = 0.0371) and uric acid levels (419.6 ± 23.91 μmol/L VS 348.3 ± 26.39 μmol/L, P = 0.0387). These findings indicate that incorporating THD into standard WM treatment for AMI provides additional benefits by further reducing inflammation, lowering uric acid levels, and enhancing kidney function.

**FIGURE 2:**
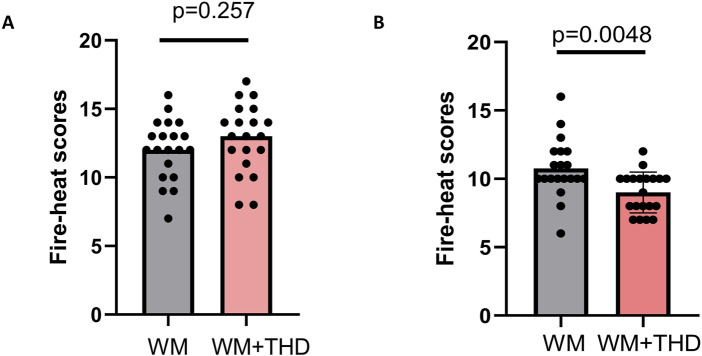
Comparison of fire-heat syndrome scores between WM and WM + THD groups. **(A)** Fire-heat syndrome scores before treatment showed no significant difference between the WM group and the WM + THD group (p = 0.257p). **(B)** Fire-heat syndrome scores after 3 days treatment demonstrated a significant reduction in the WM + THD group compared to the WM group (p = 0.0048).

Additionally, the results of the fire-heat syndrome scores (Figure 2) demonstrated significantly lower scores in the WM + THD group compared to the WM group, indicating that THD effectively alleviates fire-heat syndromes in AMI patients, such as constipation, dry mouth, yellowish urine, and a reddened tongue. This finding further suggests that THD may reduce cardiovascular burden by improving constipation, thereby potentially lowering the incidence of adverse cardiovascular events during the acute phase of AMI.

### 3.2 Proteomic analysis

**FIGURE 3:**
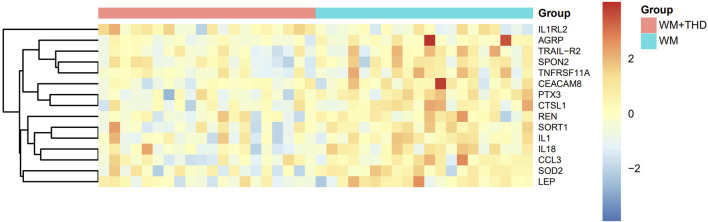
Heatmap of differentially expressed proteins between WM + THD and WM Groups. The heatmap illustrates the relative expression levels of key proteins across two groups (Group A: pink, Group B: blue). Rows represent proteins, and columns represent individual samples. The expression values are scaled and centered, with red indicating upregulation, blue indicating downregulation, and yellow representing median expression. The dendrogram on the left depicts hierarchical clustering based on protein expression profiles, showing the relationships between proteins.

**FIGURE 4:**
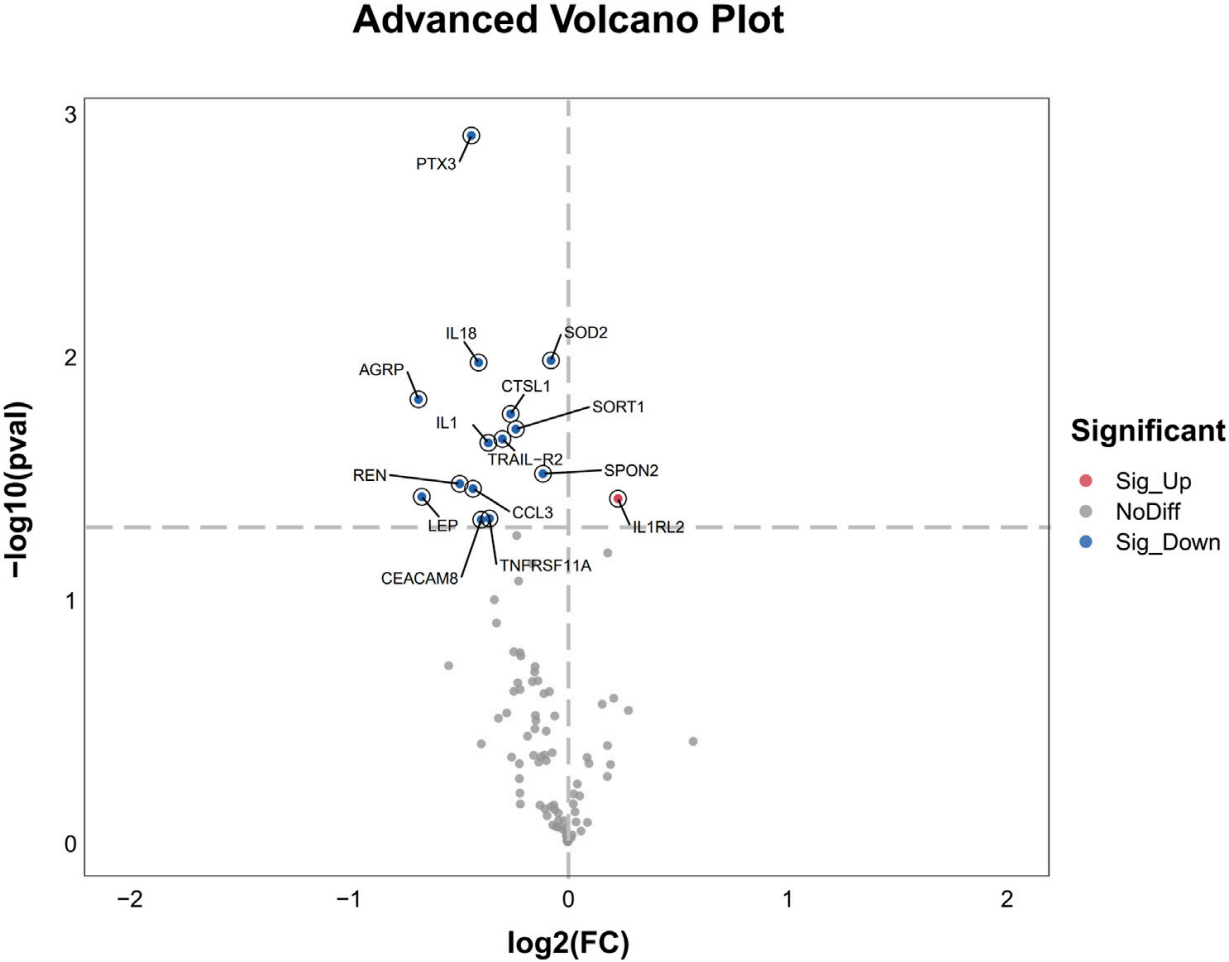
Advanced Volcano Plot of differentially expressed proteins between groups. The volcano plot displays the log2 fold change on the x-axis and the -log10 p-value on the y-axis for all analyzed proteins. Proteins with significant upregulation are highlighted in red, while significantly downregulated genes are shown in blue. Non-differentially expressed proteins are depicted in gray.

After 3 days treatment, the blood samples of patients in WM group and WM + THD group were collected to measure the proteins by the Olink^®^ Target 96 Cardiovascular III panel. Among the 96 proteins analyzed, 15 demonstrated significant differences between the two groups (Figure 3). Notably, interleukin-1 receptor-like 2 (IL1RL2) was distinctly upregulated in the WM + THD group, whereas the other 14 proteins including pentraxin-3 (PTX3), interleukin-18 (IL18), interleukin-1 (IL1), agouti-related protein (AGRP), cathepsin L1 (CTSL1), superoxide dismutase 2 (SOD2), tumor necrosis factor receptor superfamily member 11A (TNFRSF11A), TNF-related apoptosis-inducing ligand Receptor 2 (TRAIL-R2), sortilin 1 (SORT1), spondin 2 (SPON2), renin (REN), C-C motif chemokine ligand 3 (CCL3), leptin (LEP) and carcinoembryonic antigen-related cell adhesion molecule 8 (CEACAM8) exhibited consistent downregulation in this group (Figure 4). Most of the differentially expressed proteins were closely associated with inflammatory responses. IL1RL2, known for its underlying anti-inflammatory properties, was significantly upregulated following THD administration. Conversely, proteins that promote inflammation, including PTX3, IL18, IL1, CTSL1, TNFRSF11A, SPON2, CCL3, TRAIL-R2 and CEACAM8, were markedly downregulated, suggesting that THD effectively mitigates the inflammatory response triggered by AMI. Additionally, several proteins were linked to metabolic disorders such as obesity, diabetes, and hyperuricemia, including AGRP, LEP, SORT1 and SOD2. Moreover, REN was found to influence blood pressure and renal function, while TRAIL-R2 played a role in regulating apoptosis.

**FIGURE 5:**
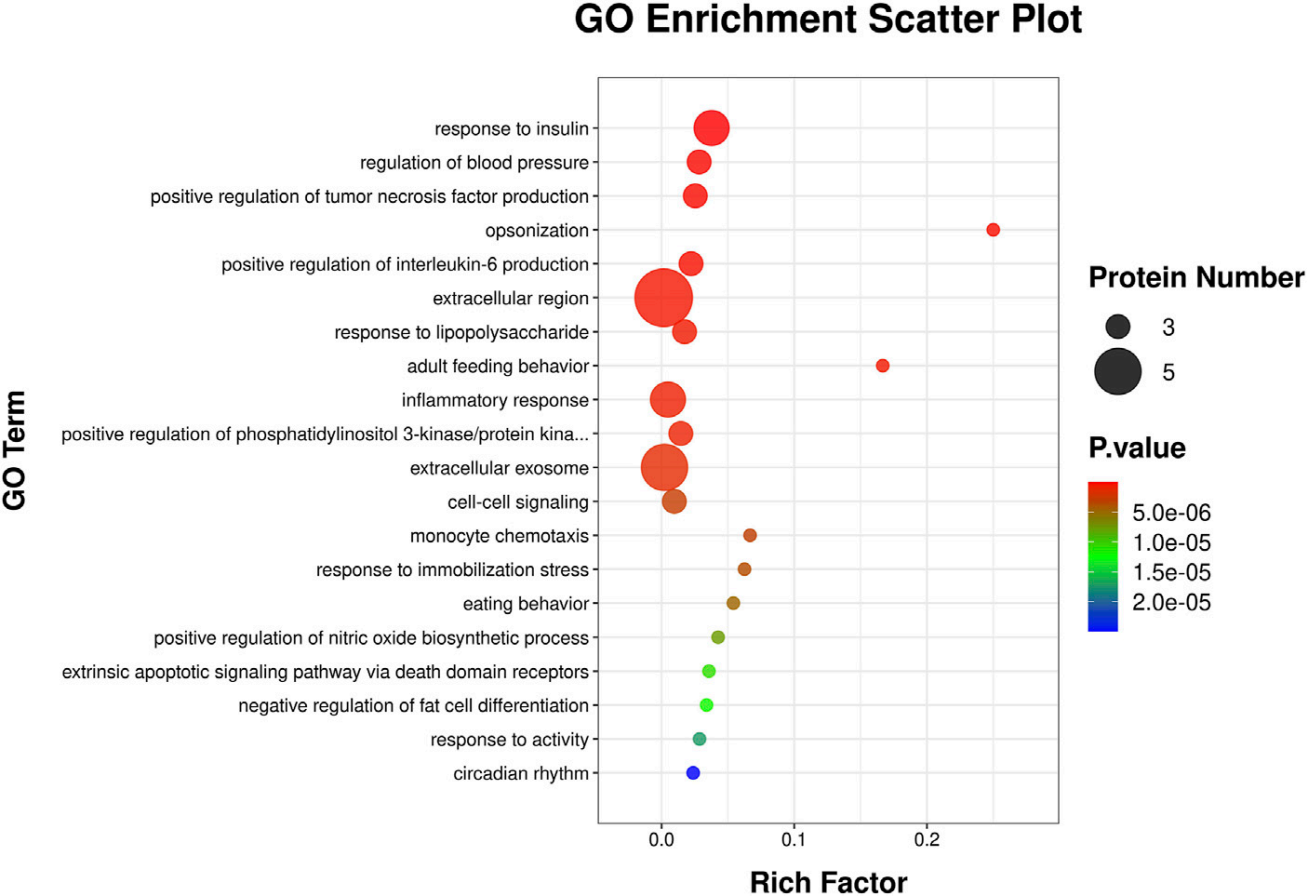
GO enrichment analysis scatter plot. The scatter plot illustrates enriched GO terms for differentially expressed genes, categorized by biological processes. The y-axis represents the GO terms, while the x-axis shows the rich factor, calculated as the ratio of differentially expressed genes to the total genes associated with the GO term. The size of each circle corresponds to the number of proteins involved in the GO term, with larger circles indicating more proteins. The color gradient represents the statistical significance of the enrichment, with red indicating higher significance and blue indicating lower significance.

This Gene Ontology (GO) enrichment scatter plot (Figure 5) visually represented the enriched biological processes, molecular functions, and cellular metabolites associated with a set of proteins. The x-axis represents the Rich Factor, defined as the ratio of proteins associated with a specific GO term to the total proteins annotated with that term in the reference set, with higher values indicating stronger associations. Circle size reflects the number of proteins linked to each GO term, where larger circles denote greater involvement. The p-value is color-coded, with darker red signifying higher statistical significance. The 20 enriched terms highlight the involvement of the analyzed proteins in key biological processes, including immune response, as indicated by terms such as “inflammatory response,” “monocyte chemotaxis,” and “positive regulation of interleukin-6 production,” reflecting strong immune and inflammatory regulatory roles. Additionally, enrichment in “response to insulin” and “negative regulation of fat cell differentiation” suggests connections to metabolic and endocrine pathways, while terms like “extrinsic apoptotic signaling pathway via death domain receptors” point to roles in cell signaling, apoptosis, and stress response.

**FIGURE 6:**
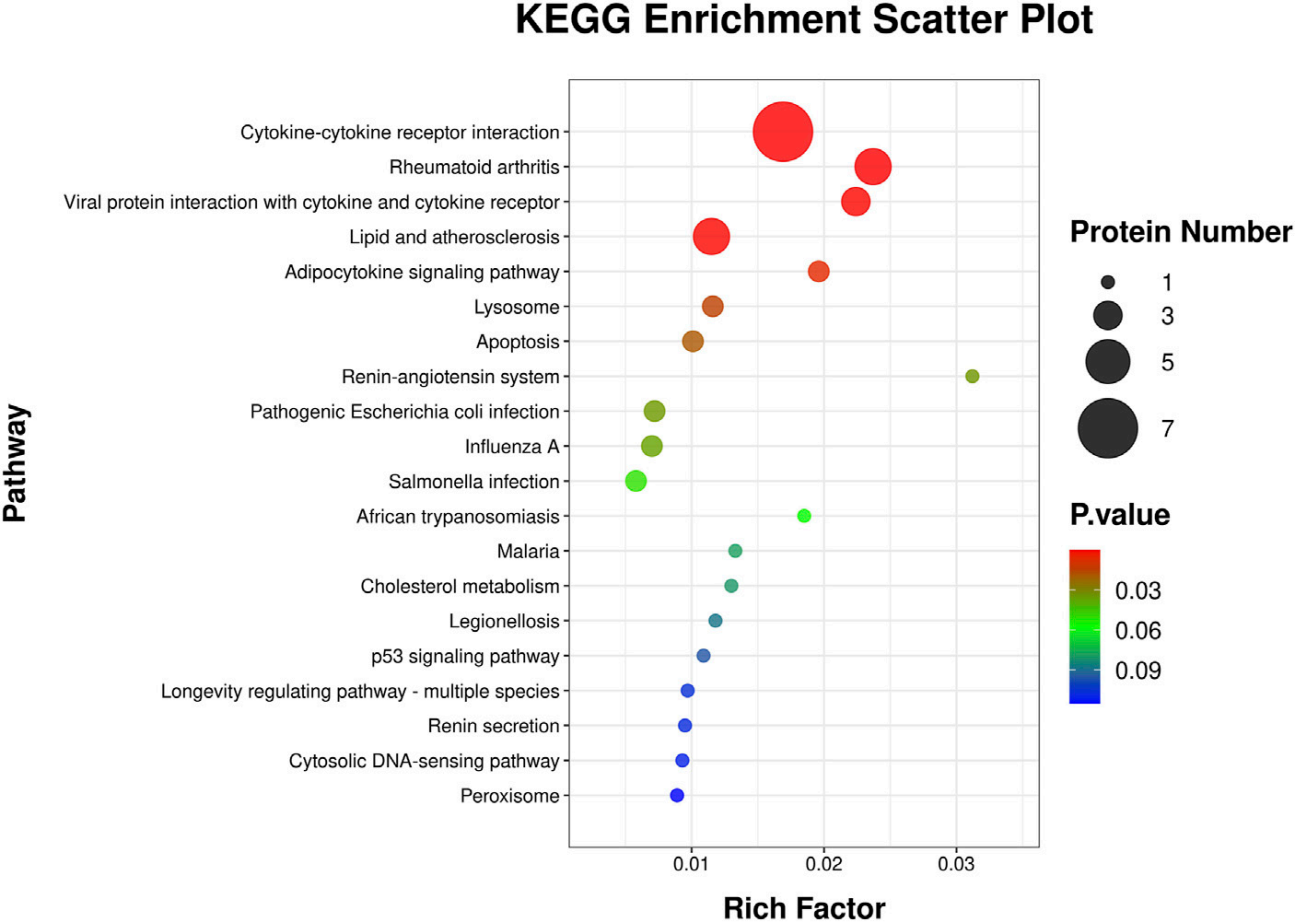
KEGG pathway enrichment analysis scatter plot. This scatter plot illustrates the KEGG pathways enriched by differentially expressed genes. The y-axis represents the KEGG pathways, while the x-axis shows the rich factor, calculated as the ratio of differentially expressed proteins to the total number of proteins associated with the pathway. The size of each circle corresponds to the number of proteins involved in the pathway, with larger circles indicating more proteins. The color gradient represents the statistical significance of the pathway enrichment, with red indicating higher significance and blue indicating lower significance.

This KEGG enrichment scatter plot (Figure 6) revealed key pathways enriched in the analyzed protein set, showcasing their involvement in diverse biological processes. The rich factor, represented on the x-axis, measures the proportion of proteins in a pathway relative to the total proteins annotated for that pathway, with higher values indicating stronger enrichment. The size of each circle reflects the protein count, with larger circles denoting a greater number of proteins associated with the pathway. Statistical significance is color-coded, with red highlighting the most significant pathways. Immune -related pathways, such as cytokine-cytokine receptor interaction, rheumatoid arthritis, and viral protein interaction with cytokine and cytokine receptor, underscore significant roles in inflammation and immune regulation. Metabolic and cardiovascular pathways, including lipid and atherosclerosis and adipocytokine signaling, highlight links to lipid metabolism, insulin sensitivity, and cardiovascular health. The enrichment of the renin-angiotensin system pathway further suggests implications for blood pressure regulation and renal function. Cellular processes like lysosome, apoptosis, and peroxisome point to critical roles in cellular degradation, programmed cell death, and oxidative stress management. Additionally, infection-related pathways such as pathogenic *Escherichia coli* infection, influenza A, and *Salmonella* infection suggest involvement in host-pathogen interactions and immune defense. Finally, pathways like the longevity regulating pathway and p53 signaling pathway connect these proteins to cellular stress responses, aging, and tumor suppression, emphasizing their broad biological significance.

These findings highlight that THD, as a traditional Chinese medicine, not only exerts potent anti-inflammatory effects but also demonstrates multi-targeted actions, influencing cardiac, renal, and metabolic pathways.

### 3.3 Network pharmacology analysis of THD

#### 3.3.1 Construction of the drug-metabolites-target network

Analysis identified 82 active metabolites of THD meeting the OB and DL criteria, distributed as follows: 15 metabolites from Huanglian (*Coptidis Rhizoma*), 16 from Dahuang (*Rhei Radix Et Rhizoma*), 22 from Lianqiao (*Forsythiae Fructus*), 14 from Dannanxing (*Arisaema Cum Bile*), and 15 from Zhuoyue (*Lophatheri Botanical druga*). Target analysis yielded 369 targets from Huanglian, 188 from Dahuang, 270 from Lianqiao, 166 from Dannanxing, and 202 from Zhuoyue. After removing duplicates, a total of 613 unique THD-related targets were identified.

**FIGURE 7:**
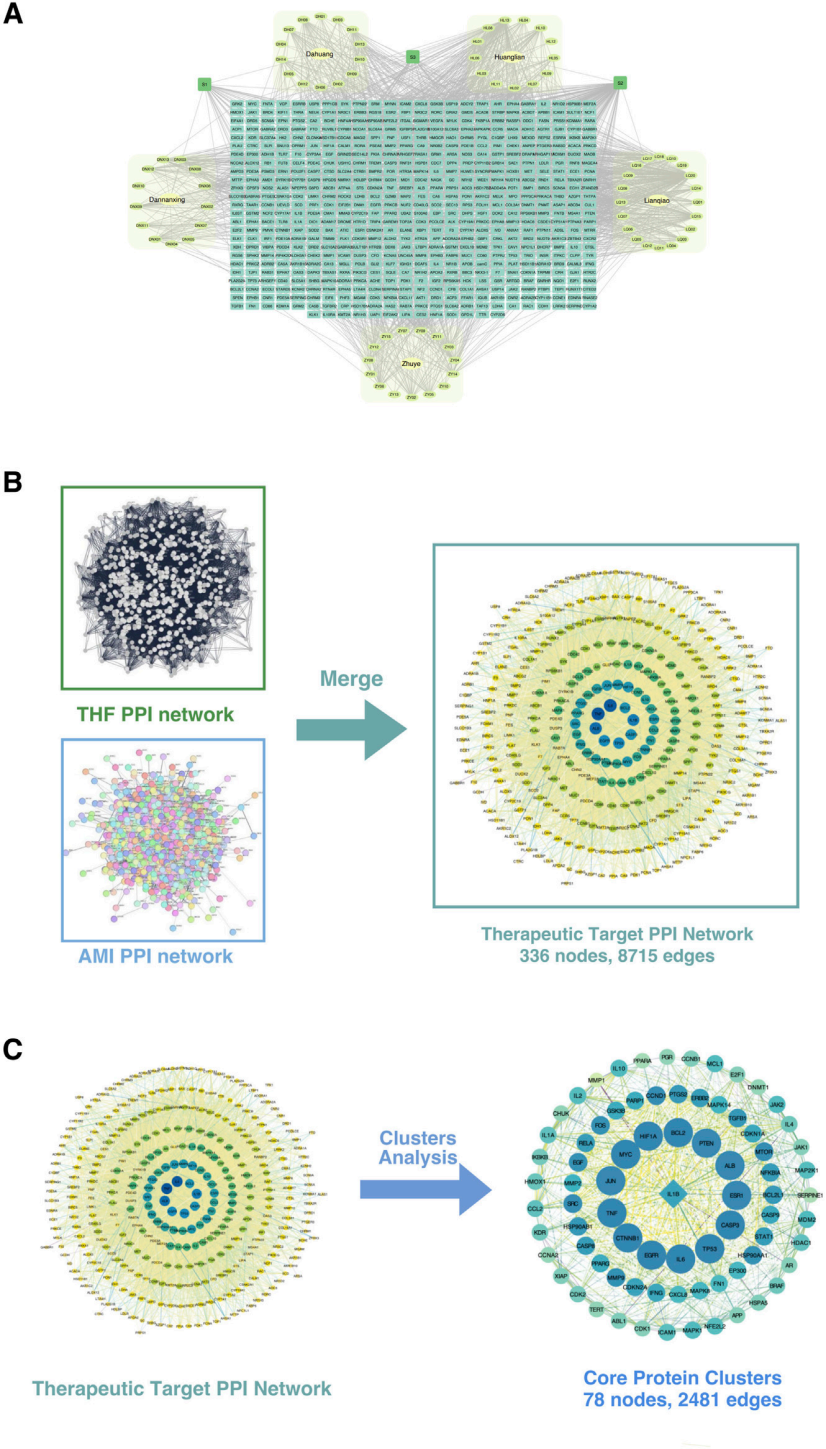
Construction and analysis of the protein-protein interaction (PPI) network. **(A)** The integrated PPI network derived from multiple datasets, including key disease-related datasets (Dunhua, Huayin, Langya, Zhou), showing interconnected nodes representing proteins and their interactions. **(B)** Merging of the THF PPI network and AMI PPI network. The THF (Traditional Herbal Formula) and AMI (Acute Myocardial Infarction) networks were combined to construct the therapeutic target PPI network, consisting of 336 nodes and 8,715 edges. **(C)** Cluster analysis of the therapeutic target PPI network. Key functional protein clusters were extracted, comprising 78 nodes and 2,481 edges. These core protein clusters represent potential therapeutic targets for the condition being studied.

The drug-metabolite-potential target network comprises 696 nodes and 1,778 edges (Figure 7A). The network includes 614 elliptical nodes representing potential therapeutic targets, 5 hexagonal nodes representing Chinese medicinal botanical drugs, and 78 circular nodes representing active metabolites. Three common metabolites were identified: Beta-Sitosterol (S1, present in Lianqiao, Dannanxing, and Dahuang), Quercetin (S2, in Huanglian and Lianqiao), and Palmidin A (S3, in Huanglian and Dahuang). The top 10 active metabolites by degree value were Quercetin (S2), Moupinamide (HL13), Kaempferol (LQ13), Luteolin (ZY09), Luteolin (LQ15), Magnograndiolide (HL02), Wogonin (LQ18), Beta-Sitosterol (S1), Procyanidin B-5,3′-O-Gallate (DH11), and Palmatine A (HL03).

#### 3.3.2 Acquisition of therapeutic targets for AMI treatment by THD

Database searches and removes duplicates yielded 2,298 unique AMI-related disease targets. Integration of the 613 drug targets and 2,298 disease targets in Cytoscape, followed by PPI network construction and merger, identified 336 intersection targets representing potential therapeutic targets of THD for AMI treatment (Figure 7B).

#### 3.3.3 Clustering analysis of therapeutic targets and construction of PPI network

The THD-AMI therapeutic target PPI network comprised 336 nodes and 8,715 edges, with node colors and sizes arranged by descending degree value. MCODE analysis identified 13 protein clusters, with Cluster 1 showing the highest score (64.442) and representing the core protein cluster. This cluster contains 78 nodes and 2,481 edges, with node colors gradating from dark to light corresponding to decreasing node degrees (Figure 7C). The top targets by degree value were BCL2, CASP3, CTNNB1, EGFR, HIF1A, IL-6, MYC, TNF, TP53, and ALB. CASP8 was identified as the Seed node, indicated by a square symbol. These targets potentially represent the key therapeutic targets of THD in AMI regulation and warrant further investigation.

#### 3.3.4 Enrichment analysis of the core protein cluster regulated by THD for AMI

**FIGURE 8:**
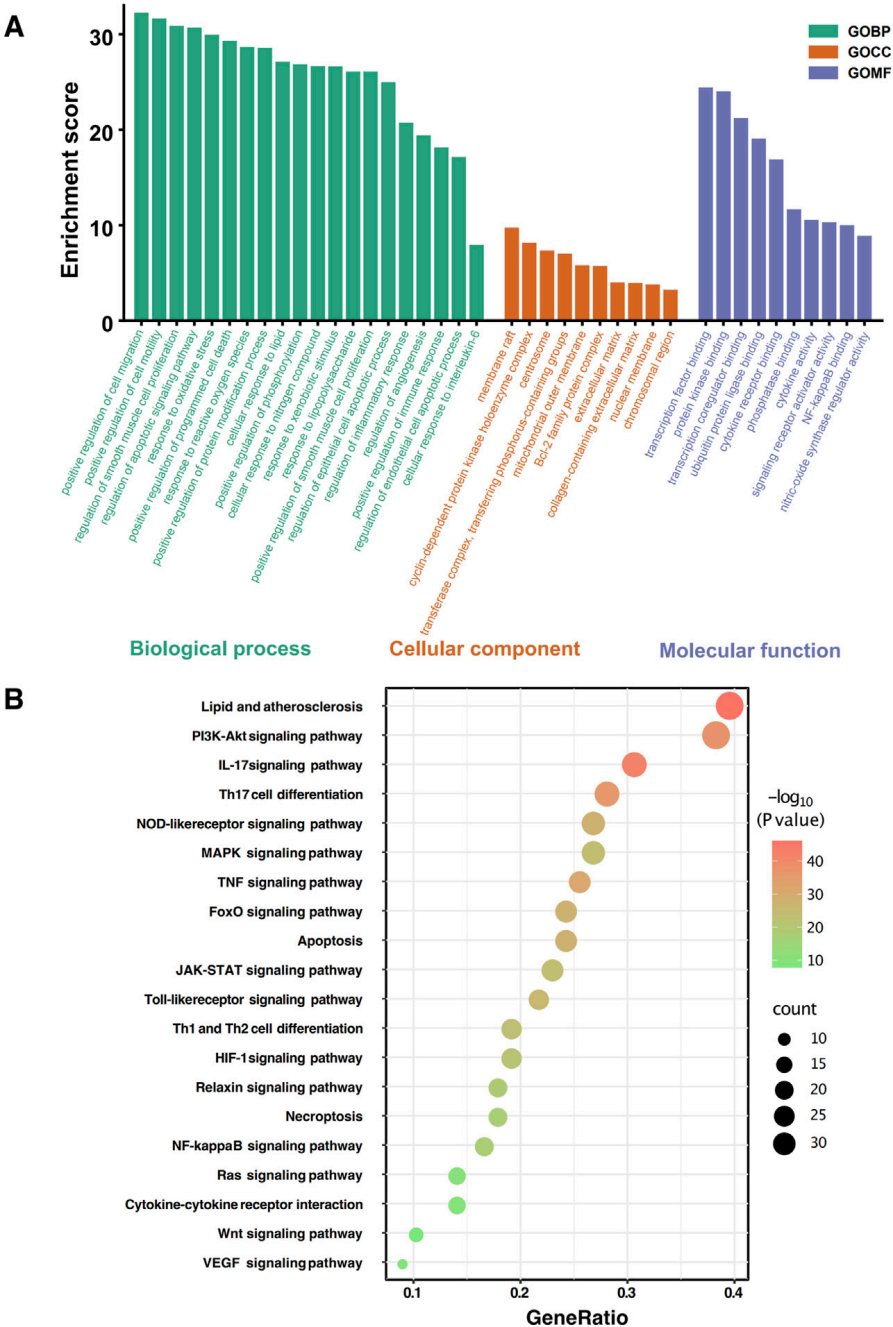
GO and KEGG Enrichment Analysis of Core Cluster Targets in AMI. **(A)** GO enrichment analysis of 78 shared targets, showing top 20 biological processes (BP), top 10 molecular functions (MF) and cellular components (CC). **(B)** KEGG pathway enrichment analysis of potential THF targets in AMI treatment, displaying top 20 pathways. Bubble size represents gene count, color indicates P-value, and x-axis shows GeneRatio.

GO and KEGG enrichment analyses revealed multiple significant pathways and processes on Metascape. The GO enrichment analysis (Figure 8A) revealed that the analyzed genes were significantly involved in key biological processes (BP), such as the positive regulation of tumor necrosis factor production, response to insulin, regulation of blood pressure, and inflammatory response, underscoring their roles in immune regulation, inflammation, and metabolic control. The cellular metabolite (CC) analysis highlighted their localization within the extracellular region, plasma membrane receptor complex, and nuclear transcription factor complex, reflecting their involvement in signal transduction and extracellular communication. Furthermore, the molecular function (MF) analysis emphasized their roles in immune signaling, receptor interactions, and oxidative stress regulation, as evidenced by enriched functions such as cytokine receptor binding, signaling receptor activator activity, and oxidoreductase activity.

The KEGG enrichment scatter plot (Figure 8B) highlighted key pathways associated with the analyzed proteins, reflecting their diverse biological roles. Among the top-enriched pathways were lipid and atherosclerosis, PI3K-Akt signaling, and IL-17 signaling, emphasizing their involvement in cardiovascular health, immune responses, and inflammation. Immune regulation and inflammatory signaling were further underscored by pathways such as Th17 cell differentiation, Toll-like receptor signaling, and TNF signaling. Additionally, enrichment in pathways like MAPK signaling, FoxO signaling, JAK-STAT signaling, and apoptosis highlighted their critical roles in cellular stress responses, survival, and programmed cell death. Metabolic and vascular-related pathways, including HIF-1 signaling, relaxin signaling, VEGF signaling, and Wnt signaling, further illustrated their contribution to vascular development, hypoxia adaptation, and tissue remodeling, showcasing the broad biological significance of these proteins. These pathways were visualized in a bubble chart where the vertical axis represented pathway names, the horizontal axis represented generation, node size indicated gene count, and node color represented P-value.

## 4 Discussion

Our study highlights the significant therapeutic potential of THD in managing AMI) by addressing the interconnected cardiorenal pathophysiology through multi-targeted anti-inflammatory and metabolic modulation. AMI-induced myocardial necrosis and systemic inflammatory responses often result in cardiac dysfunction, heart failure, and renal perfusion deficits, exacerbated by systemic responses such as sympathetic overactivation and renin-angiotensin-aldosterone system upregulation. While conventional treatments targeting singular mechanisms fail to fully mitigate residual inflammatory and metabolic risks, THD uniquely demonstrates the ability to reduce inflammation, lower hs-CRP and pro-inflammatory cytokine levels, and improve metabolic parameters such as uric acid. This dual mechanism alleviates oxidative stress, protects the myocardium, and enhances renal function, offering a comprehensive strategy to reduce the long-term risks of heart failure and chronic kidney disease. These findings underscore the importance of early identification of high-risk individuals and targeted interventions to optimize clinical outcomes and mitigate systemic complications in AMI management.

Our results indicated that AMI patients receiving WM treatment alone exhibited a sustained increase in hs-CRP and uric acid levels. In contrast, patients treated with WM + THD demonstrated a significant reduction in hs-CRP and uric acid. Additionally, compared to the WM group, patients in the WM + THD group showed a marked increase in CCR and a significant decrease in inflammatory markers such as neutrophil count and hs-CRP. These findings suggest that THD may exert cardiorenal protective effects by alleviating the inflammatory response. Furthermore, as a traditional Chinese medicine, THD is primarily used to treat patients with fire-heat syndrome, characterized by higher fire-heat scores indicating more severe symptoms, including constipation, dry mouth, yellow urine, and reddened tongue coating. Our study revealed that patients treated with WM + THD exhibited significantly lower fire-heat scores compared to those receiving WM alone. This finding further demonstrates that THD effectively alleviates fire-heat symptoms, particularly by improving constipation, thereby reducing myocardial burden and protecting cardiac and renal function in patients with AMI.

### 4.1 Potential cardioprotective mechanisms of THD

A substantial body of research has established that inflammation is a major determinant of poor prognosis in AMI, serving as an independent risk factor for heart failure and cardiac death (Weber et al., 2023; Priscilla et al., 2024; Ridker et al., 2023; Zhang et al., 2024; Guo et al., 2024). Mitigating residual inflammatory risk through inflammation reduction is crucial to improving outcomes in AMI patients. However, Western medications often fall short of achieving the desired prognostic benefits in AMI patients due to their single-target approach and significant side effects (Matter et al., 2024). In contrast, TCM has garnered increasing attention for its multi-target mechanisms and minimal adverse effects.

Proteomic analysis revealed that, compared to the WM group, IL1RL2 recognized for its inherent anti-inflammatory potential, exhibited substantial upregulation after THD treatment. In contrast, key pro-inflammatory proteins such as PTX3, IL-18, IL-1, CTSL1, TNFRSF11A, SPON2, CCL3, TRAIL-R2 and CEACAM8 showed pronounced downregulation. IL1RL2, a key metabolite of the interleukin-1 receptor family, functions as the binding receptor for IL-36 cytokines, including IL-36α, IL-36β, and IL-36γ (Manzanares-Meza et al., 2022). The IL-36/IL1RL2 axis activates the nuclear factor kappa-light-chain-enhancer of activated B cells (NF-κB) and mitogen activated protein kinase (MAPK) signaling pathways, driving inflammatory responses (Queen et al., 2019). However, elevated levels of IL1RL2 may attenuate excessive inflammatory signaling by upregulating anti-inflammatory cytokines or functioning as a “decoy receptor”. Furthermore, relevant studies have demonstrated that IL1RL2 signaling can activate intestinal epithelial cells and fibroblasts, facilitating mucosal healing *in vivo*. This suggests that IL1RL2 may also play a role in promoting cardiac repair following AMI (Scheibe et al., 2017).

PTX3 has been identified as an early marker of vascular inflammation and endothelial dysfunction, notably in conditions such as AMI and atherosclerosis (Zhao et al., 2023). It is known to amplify the inflammatory response by facilitating the recruitment of immune cells to sites of vascular injury or dysfunction. IL-1 and IL-18, as key pro-inflammatory cytokines, exacerbate myocardial damage in AMI through their roles in amplifying the inflammatory response. IL-1, particularly IL-1β, triggers the activation of NF-κB signaling, leading to the recruitment of immune cells, increased production of inflammatory mediators, and enhanced cardiomyocyte apoptosis and necrosis (Buckley and Abbate, 2018). Similarly, IL-18 promotes Th1-driven inflammation by inducing IFN-γ production, aggravating oxidative stress, endothelial dysfunction, and myocardial injury (O'Brien et al., 2014). CTSL1 was implicated in activating pro-inflammatory signaling pathways, amplifying the inflammatory response in the ischemic myocardium and it has been reported that it was positively correlated with the risk of mortality in AMI (Wu et al., 2022).

CCL3, also known as macrophage inflammatory protein-1 alpha (MIP-1α), is a chemokine that plays a critical role in recruiting and activating immune cells during inflammatory responses and it is recognized as a contributing factor to poor prognosis in acute coronary syndrome (ACS) (de Jager et al., 2008). TNFRSF11A, also called receptor activator of nuclear factor κ B (RANK), is a member of the tumor necrosis factor receptor superfamily playing a pivotal role in activating the NF-κB signaling pathway to promote inflammatory response (Raju et al., 2011). SPON2, also referred to as Mindin, is a glycoprotein found in the extracellular matrix that plays a crucial role in modulating immune responses and driving inflammation. By activating immune cells, SPON2 enhances their pro-inflammatory activity, stimulating the release of cytokines and chemokines that intensify the inflammatory cascade (Huang et al., 2021). CEACAM8, or CD66b, is a glycoprotein primarily found on neutrophils. When activated, these neutrophils release pro-inflammatory cytokines and chemokines, intensifying the immune response and contributing to the amplification of inflammation (Ribon et al., 2019). TRAIL-R2, a member of the tumor necrosis factor receptor superfamily, primarily induces apoptosis through its interaction with the ligand TRAIL. Studies have revealed that the TRAIL/TRAIL-R2 axis not only directly triggers cardiomyocyte apoptosis but also recruits and activates inflammatory cells, promoting the release of pro-inflammatory cytokines (Wang et al., 2020). This exacerbates myocardial injury, establishing TRAIL-R2 as a positive marker associated with adverse outcomes in AMI (Wang et al., 2020; Kuang et al., 2023).

Moreover, proteomic and network pharmacology analyses, including GO and KEGG results, revealed that THD engaged in inflammatory pathways through multiple mechanisms, highlighting its multi-target pharmacological effects. Above all the results illuminated that THD mitigated residual inflammatory risk by attenuating inflammation, thereby providing cardioprotective effects for patients recovering from AMI. This intervention may reduce the incidence of adverse cardiovascular events such as heart failure and mortality after discharge, enhancing long-term outcomes.

### 4.2 Underlying renal protective mechanisms of THD

Renal function decline in AMI is profoundly intertwined with inflammatory responses, as the aforementioned inflammatory proteins and pathways, particularly the NF-κB and MAPK signaling pathways, contributing to renal dysfunction (Ren et al., 2020; Li S. et al., 2023; Li J. et al., 2023). Furthermore, elevated uric acid (UA) levels can exacerbate inflammatory responses, significantly increasing the risk of cardiac and renal dysfunction (Li et al., 2023c; Saito et al., 2021). Oxidative stress, characterized by an overproduction of reactive oxygen species (ROS), is one of the key contributors to renal function decline in AMI. While UA possesses antioxidant properties under normal conditions, elevated levels can paradoxically increase ROS generation, exacerbating oxidative stress and causing damage to renal tubular epithelial cells (Miake et al., 2023). SOD2 facilitates the conversion of superoxide radicals (O_2_^−^) into hydrogen peroxide (H_2_O_2_) and oxygen (O_2_), thereby alleviating oxidative stress within mitochondria. Proteomic analysis in this study revealed a decrease in SOD2 expression in the WM + THD group. This reduction may be attributed to THD’s ability to lower UA levels and mitigate inflammatory responses, thereby alleviating oxidative stress. Consequently, the relative expression of SOD2 was reduced compared to the WM group.

Renin, a pivotal enzyme in the renin-angiotensin system (RAS), not only regulates blood pressure but can also exacerbate renal injury by inducing vasoconstriction, reducing perfusion, and amplifying oxidative stress and inflammation (Ma et al., 2022). In this study, the downregulation of REN expression following THD treatment further substantiates THD’s multi-targeted protective effects on renal function.

### 4.3 The potential metabolic impacts of THD

Proteomic analysis illustrated that the expression of certain proteins including AGRP, LEP and SORT1 associated with metabolic diseases decreased following THD treatment. AGRP has been shown to stimulate appetite, diminish energy expenditure, and contribute to obesity, while also elevating the risk of adverse cardiovascular outcomes in the context of AMI (Liu et al., 2023). Leptin, a hormone secreted by adipose tissue, is closely associated with diabetes and other metabolic disorders. Studies have demonstrated that leptin levels are elevated in the subcutaneous adipose tissue of prediabetic individuals, where it promotes inflammatory responses and increases the risk of cardiovascular diseases (Sardu et al., 2019). SORT1, a type I transmembrane receptor from the Vps10p protein family, plays a crucial role in lipid metabolism and cardiovascular health. It regulates low-density lipoprotein (LDL) and very low-density lipoprotein (VLDL) metabolism in hepatocytes, influencing processes such as VLDL secretion, LDL clearance, and foam cell formation. Aberrant SORT1 expression contributes to atherosclerosis by promoting lipoprotein imbalance, arterial inflammation, and vascular calcification (Kim et al., 2023). Moreover, proteomic and network pharmacology analyses, as reflected in the GO and KEGG results, demonstrated THD’s involvement in diverse metabolic pathways, notably those related to lipid and glucose regulation. These findings underscore THD’s potential to influence metabolic processes, further accentuating its multi-targeted mechanisms and its protective effects on cardiac function.

### 4.4 The theoretical basis of treating AMI from fire-heat in TCM

AMI is categorized in TCM as “Xin Tong” (heart pain) and “Zhen Xin Tong” (true heart pain), with core pathogenesis involving qi stagnation, blood stasis, phlegm obstruction, and excessive fire, where fire-heat plays a key role in disease progression. From a biomedical perspective, AMI triggers a strong inflammatory response, activating cytokines such as IL-6, TNF-α, and CRP, which contribute to myocardial injury and necrosis (Nah and Rhee, 2009). TCM associates excessive inflammation with “intense heat toxin” and “reckless movement of hot blood”, exacerbating blood stasis. Studies suggest that heat-clearing, detoxifying, and blood-activating botanical drugs (e.g., Coptis chinensis, Gardenia jasminoides, Salvia miltiorrhiza, Paeonia veitchii) can reduce CRP and TNF-α levels, alleviating myocardial injury (Wu et al., 2016; Chen et al., 2020; Zhou et al., 2012; Ren et al., 2010; Li et al., 2016). Formulas such as Guan-Xin-Er-Hao and Danshen Yin further suppress AMI-related inflammatory cytokines and improve endothelial function (Ma et al., 2022; Liu et al., 2023; Sardu et al., 2019; Kim et al., 2023; Nah and Rhee, 2009; Wu et al., 2016; Chen et al., 2020; Zhou et al., 2012; Ren et al., 2010; Li et al., 2016; Zhang et al., 2010; Yan et al., 2012). Additionally, the THD in our previous study has shown efficacy in reducing TNF-α and IL-6 levels, highlighting TCM’s potential anti-inflammatory benefits in AMI (Nie et al., 2022). However, further mechanistic studies and clinical trials are needed to confirm these effects.

### 4.5 Limitations

Despite its promising findings, this study has some limitations: Firstly, the randomized controlled trial included only 40 participants, which may limit the generalizability of the results. A larger cohort is needed to validate the observed therapeutic effects of THD; Secondly, the intervention lasted only 3 days, focusing on immediate effects. We will further verify the long-term impact of THD on major adverse cardiac events, inflammatory markers, as well as metabolic indicators such as uric acid, body weight, and blood glucose through follow-up studies; Finally, our mechanistic study is primarily based on network pharmacology and proteomics, and further experimental validation is essential. Future research should include animal models of AMI to assess THD’s effects on inflammatory pathways (NF-κB, IL-1β, TNF-α, IL-18) and metabolic regulation (lipid metabolism, insulin sensitivity). Additionally, *in vivo* studies should evaluate THD’s impact on myocardial infarct size, cardiac output, and renal function markers. Furthermore, with regard to fire-heat syndrome, our study employed only a preliminary symptom scoring system, lacking a comprehensive TCM syndrome differentiation that includes detailed assessments of yellow tongue coating and pulse characteristics. In future research, we will implement stricter inclusion criteria to specifically enroll AMI patients with concurrent fire-heat syndrome and systematically evaluate the therapeutic effects of THD on fire-heat syndrome manifestations.

## 5 Conclusion

THD exerts cardiorenal protection by regulating inflammation and metabolic signaling pathways through multiple targets, demonstrating great therapeutic potential in the treatment of AMI. Its ability to address the interconnected pathophysiological mechanisms of AMI underscores its value as a traditional medicine with modern clinical relevance. Further research and clinical validation are warranted to fully harness its therapeutic benefits.

## Data Availability

The data that support the findings of this study are available on request from the corresponding authors.
