# Supplementary material for: Cardiorenal protective effects of Tanhuo decoction in acute myocardial infarction via regulating multi-target inflammation and metabolic signaling pathways
Source: Front Pharmacol. 2025 Mar 27;16:1555605. doi: 10.3389/fphar.2025.1555605 (PMC12000776; doi:10.3389/fphar.2025.1555605)
Supplement: Supplementary file 1 [file Table1.docx]

**Supplementary Table 1.** Batch numbers and components of the herbs in THD.

| Herb | Information |
| --- | --- |
| Rhei Radix et Rhizoma  (Batch number: DD1251) | **Components:**  The total content of free anthraquinone $\geq$ 0.35%, including aloe emodin (C_15_H_10_O_5_), rhein acid (C_15_H_8_O_6_), emodin (C_15_H_10_O_5_), chrysophanol (C_15_H_10_O_4_), and emodin methyl ether (C_16_H_12_O_5_).  **Extracts:**  $\geq$ 25%;  The total content of anthraquinone $\geq$1.5%, including aloe emodin (C_15_H_10_O_5_), rhein acid (C_15_H_8_O_6_), emodin (C_15_H_10_O_5_), chrysophanol (C_15_H_10_O_4_), and emodin methyl ether (C_16_H_12_O_5_).  **Examination:**  SO_2_ residue $\leq$ 150mg/kg;  Rhubarb glycoside: in the chromatogram of the test sample, the same bright blue fluorescent spots shall not be shown on the position corresponding to the reference sample of rhubarb glycoside;  Dry weight loss $\leq$ 15.0%;  Total ash content $\leq$ 10.0%. |
| Coptidis Rhizoma  (Batch number: DC6061) | **Components:**  Berberine (C_20_H_17_NO_4_) $\geq$ 5.0%;  The total content of berberine (C_20_H_17_NO_4_), coptisine (C_19_H_13_NO_4_) and palmatine (C_21_H_21_NO_4_) $\geq$ 3.3%.  **Extracts:**  $\geq$ 15.0%;  **Examination:**  SO_2_ residue $\leq$150mg/kg;  Moisture $\leq$ 12.0%;  Total ash content $\leq$ 3.5%;  Auramine O: in the chromatogram of the test sample, the spots with the color same as that of Auramine O shall not be shown on the position corresponding to the reference sample of Auramine O. |
| Forsythia  (Batch number: DD1161) | **Components:**  Forsythin (C_27_H_34_O_11_) $\geq$ 0.15%;  Forsythiasin A(C_29_H_36_O_15_) $\geq$ 0.25%.  **Examination:**  SO_2_ residue $\leq$ 150mg/kg;  Unripe forsythia suspense $\leq$ 3%;  Moisture $\leq$ 10.0%;  Total ash content $\leq$ 4.0%. |
| Bile Arisaema  (Batch number: DD3081) | **Examination:**  SO_2_ residue $\leq$ 150mg/kg;  Total ash content $\leq$ 10.0%. |
| Lophatherum Gracile  (Batch number: DC7061) | **Examination:**  SO_2_ residue $\leq$ 150mg/kg;  Moisture $\leq$ 13.0%;  Total ash content $\leq$ 11.0%. |

Note: bold, category of the information of the herb.
