# Supplementary figures and images for: Cardiorenal protective effects of Tanhuo decoction in acute myocardial infarction via regulating multi-target inflammation and metabolic signaling pathways
Source: Front Pharmacol. 2025 Mar 27;16:1555605. doi: 10.3389/fphar.2025.1555605 (PMC12000776; doi:10.3389/fphar.2025.1555605)

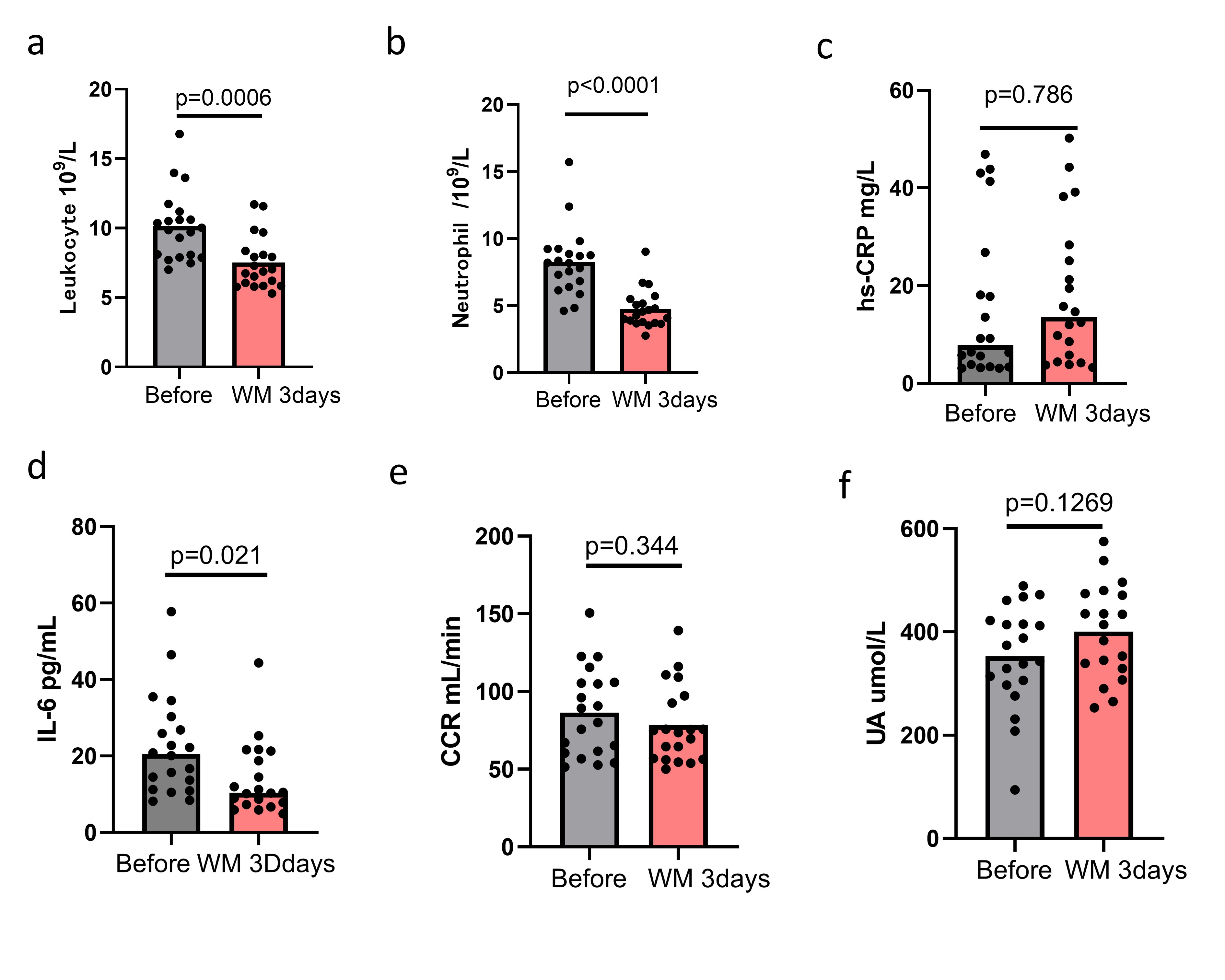

Supplement: Supplementary file 2 [file Image1.jpeg]

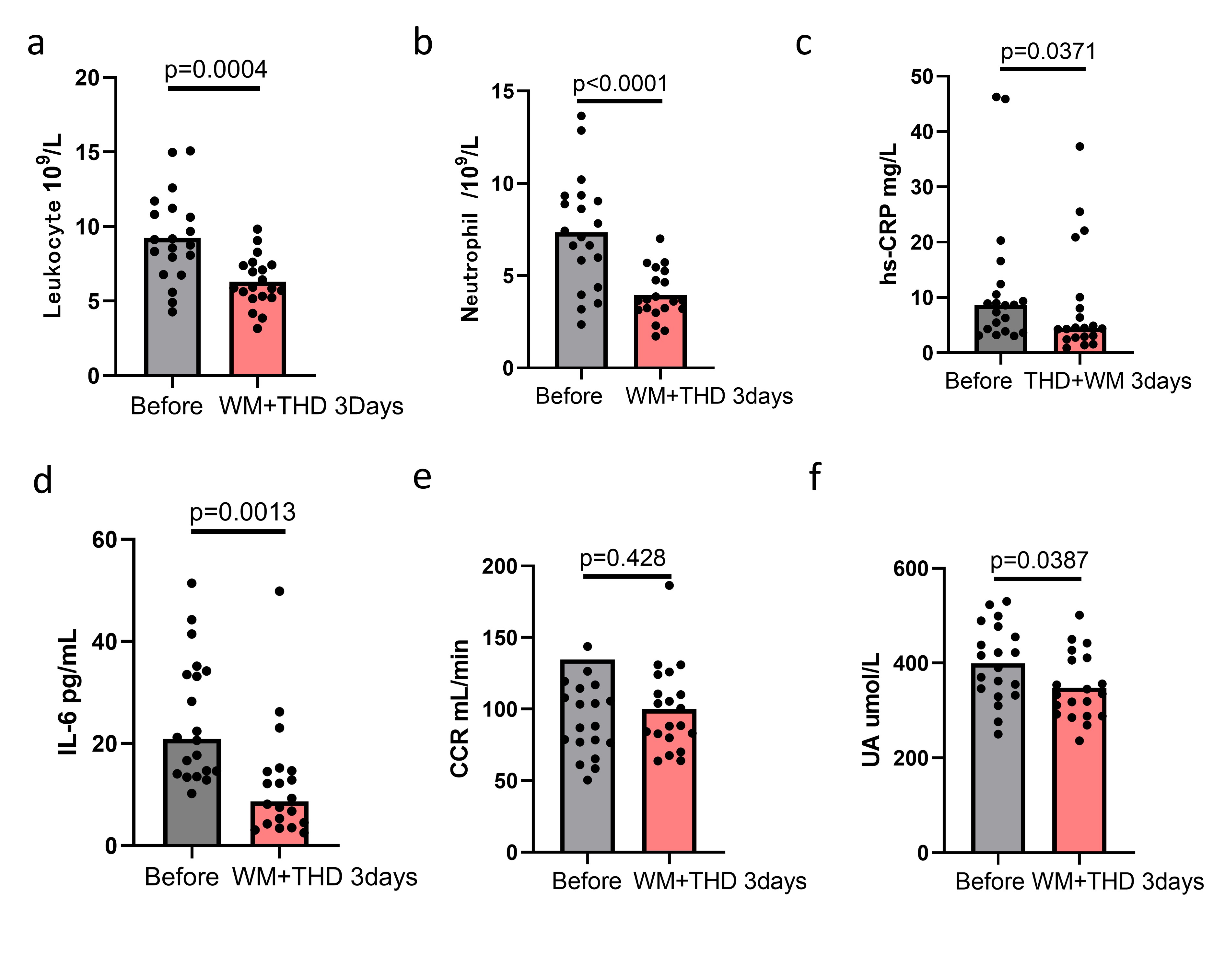

Supplement: Supplementary file 3 [file Image2.jpeg]
